# Validation of the Center for Epidemiologic Studies Depression Scale (CES-D) in a Moroccan sample with substance use disorder

**DOI:** 10.1186/s12888-023-05245-2

**Published:** 2023-10-06

**Authors:** Abdelfettah El-Ammari, Hicham El Malki, Salma Ghofrane Moutawakkil, Jaouad El Hilaly, Fatima El Houari, Samir El Gnaoui, Mohammed El Amine Ragala, Karima El Rhazi, Btissame Zarrouq

**Affiliations:** 1 Laboratory of Epidemiology and Research in Health Sciences, Faculty of Medicine, Pharmacy, and Dental Medicine, Sidi Mohamed Ben Abdellah University, Fez, Morocco; 2 R.N.E Laboratory, Multidisciplinary Faculty of Taza, Sidi Mohamed Ben Abdellah University, Fez, Morocco; 3 Laboratory of Pedagogical and Didactic Engineering of Sciences and Mathematics, Regional Center of Education and Training (CRMEF) of Fez, Fez, Morocco; 4 Addictology Center of Fez, Fez, Morocco; 5 Department of Biology-Geology, Teachers Training College (Ecole Normale Superieure), Sidi Mohamed Ben Abdellah University, Fez, Morocco

**Keywords:** Psychometric quality, Convergent validity, Discriminant validity, Reliability, Composite reliability, Substance use disorder, Depression, Center for Epidemiologic Studies Depression Scale (CES-D)

## Abstract

**Background:**

Transcultural validation studies of depression scales are rare in Morocco. The Center for Epidemiologic Studies Depression Scale (CES-D) is commonly one of the most common and frequently used screening instruments for depressive symptoms, but the scale has not, up to date, been validated in dialect of Arabic in Moroccan contexts. Given the importance of assessing and preventing depressive symptoms in our Moroccan context, this study aims to validate the CES-D, translated, and adapted to the dialect of Arabic and Moroccan culture, in a sample with substance use disorder.

**Methods:**

The data were analyzed in two successive phases. First, exploratory factor analysis (EFA) was used to assess the factor structure in the pilot sample (*N* = 140). Then, this structure was confirmed in the validation sample (*N* = 205) using confirmatory factor analysis (CFA).

**Results:**

Exploratory factor analysis extracted three factors different from the four factors in the original version. Confirmatory factor analysis confirmed the structure of three factors. The fit indices level showed acceptable to good performance of the measurement model. The instrument showed sufficient reliability and convergent validity, as demonstrated by acceptable values of composite reliability (CR = 0.89–0.93) and average variance extracted (AVE = 0.64–0.66), respectively. The square roots of AVE were higher than factor-factor pairs correlations, and the Heterotrait-Monotrait ratio (HTMT) of correlations values was less than 0.85, indicating acceptable discriminant validity.

**Conclusions:**

Overall reliability and both convergent and discriminant validity tests indicated that the Moroccan dialectal Arabic version of the CES-D had a good performance and may serve as a valid tool for measuring the severity of depression in people with substance use disorder.

## Background

Mental disorders can lead to significant physical, emotional, and social health issues, and can greatly impact the daily lives of those affected [1, 2]. They are a significant contributor to the global burden of disease, disability, illness, and death [3–5]. In total, poor mental health was estimated to cost the global economy about $2.5 trillion annually in ill health and productivity losses in 2010, with a projected cost of $6 trillion by 2030 [6]. According to the 2019 Global Burden of Disease, Injury, and Risk Factors Study, they ranked among the top 25 causes of stress globally in 2019 [1, 7]. In 2020, the COVID-19 pandemic led to a significant rise in the number of people experiencing such disorders. Early estimates indicate a one-year increase of 28% and 26% for major depressive disorders and anxiety disorders, respectively [1].

The co-occurrence of mental disorders and substance use disorders (SUD) compounds the existing challenges in mental health; indeed, substance use disorders also have a high prevalence among individuals with mental disorders [8–11]. This comorbidity not only affects a substantial portion of the global population but also contributes significantly to the burden of disease, accounting for 7% of the total global disability-adjusted life years (DALYs). In 2016, they affected over 1 billion people worldwide. They caused 19% of all years lived with disability. Depression, a prevalent mental health condition, holds a central position in this context [12]. It ranks highest in terms of DALYs among both men and women and exhibits a higher prevalence in women compared to other internalizing disorders. Conversely, SUD exhibits a higher prevalence among men [3, 13].

Depression, as defined by the Diagnostic and Statistical Manual of Mental Disorders, 5th Edition (DSM-V), is characterized by a constellation of symptoms, including sadness, loss of interest in or pleasure in nearly all activities, and other physical and psychological symptoms. These symptoms may include changes in appetite or weight, sleep disturbances, fatigue, feelings of worthlessness or excessive guilt, difficulty concentrating, and recurrent thoughts of death or suicide. The DSM-V requires the presence of at least five of these symptoms for a minimum duration of two weeks in order to diagnose major depressive disorder. These symptoms must also cause significant impairment in social, occupational, or other areas of functioning [14].

Untreated depression can persist for an extended period and impact daily activities, such as academic and social functioning [15]. Depression can lead to drug abuse and suicide in severe cases [16–19]. Estimates consistently show that a significant portion of individuals with substance abuse issues also experience symptoms of depression. People who have comorbid depression and SUD tend to experience more severe symptoms, greater disability, and higher rates of hospitalization and mortality. Treatment outcomes are also poor for patients who have both of these conditions. It has a high dropout rate, a low rate of symptom reduction, and a high rate of relapse [20–26].

According to the World Health Organization (WHO), depression affects a staggering 280 million people worldwide, making it the primary cause of disability globally and a significant risk factor for suicide. According to estimates, depression affects 3.8% of the population, including 5% of adults (4% of males and 6% of females) and 5.7% of individuals over the age of 60 [12].

Depression's prevalence varies across countries and regions globally. A study conducted in 18 countries (with a sample size of 89,037) found that the average lifetime and 12-month prevalence estimates of major depressive episodes were 14.6% and 5.5% in the 10 high-income nations, respectively, and 11.1% and 5.9% in the eight low- to middle-income nations, respectively. In high-income countries, the average age of onset is 25.7, while in low- to middle-income nations, it is 24.0 [27]. Experts predict that by 2030, depression alone is likely to become the third leading cause of disease burden in low-income countries and the second highest cause of disease burden in middle-income countries [28].

Morocco, a low- and middle-income country, grapples with the significant health challenge posed by depression [29]. Regrettably, acquiring reliable data on both drug use and mental health in Morocco proves to be a daunting task. To date, the only comprehensive national survey, conducted in 2005 by the Ministry of Health among a sample of 6,000 individuals aged 15 and older, estimated the prevalence of depression to be 26.5%. It was more frequently observed in women (34.3%) than in men (20.4%) [30]. In contrast, a survey published in 2020 by the Haut Commissariat au Plan (HCP) reported a lower prevalence rate of 5.9% for depression [31]. Regarding the use of psychoactive substances, the Ministry of Health's study found that the annual prevalence of illegal drug use in Morocco among the adult general population was 4.1%, with cannabis alone representing 3.93% of this total. Substance abuse was reported at 3.0%, and substance dependence at 2.8%. Specifically, alcohol abuse stood at 2.0%, and alcohol dependence at 1.4% [30]. Except for this study, the majority of other studies primarily focus on young individuals, for example, the most significant survey, MedSPAD (Mediterranean School Project on Alcohol and other Drugs), which is a Moroccan adaptation of the European survey ESPAD (European School Survey Project on Alcohol and other Drugs), aims to monitor evolution in drug consumption among young people. Conducted in 2009, 2013, and 2017, this study reveals that within the same age group, there is a slight increase in prevalence figures between the 2013 and 2017 MedSPAD surveys for substances such as tobacco, alcohol, cannabis, and benzodiazepines. In the 2017 MedSPAD survey, the annual prevalence rates for the most consumed substances were as follows: for tobacco (12.6% for boys and 2.2% for girls), for cannabis (12.0% for boys and 1.2% for girls), and for alcohol (6.9% for boys and 1.0% for girls) [32]. Two other studies conducted at the regional level in Morocco. The first one, carried out in the North-Central region of Morocco from April 2012 to November 2013 among 3,020 students, showed that the prevalence of current smokers was 9.1%, with an overall lifetime prevalence of psychoactive substance consumption at 9.4% [33]. Cannabis had the highest lifetime prevalence at 8.08%, followed by alcohol at 4.31%. In 2020, the second study conducted in the Beni Mellal region reported an overall prevalence of different psychoactive substance use among students estimated at 20.6% [34]." In Morocco, the comorbidity between substance use disorders and depression is particularly pronounced, as some individuals turn to psychoactive substances like cannabis to cope with their depression. Cannabis is widely available in Morocco due to traditional cultivation practices, and its abusive use can exacerbate mental health problems, perpetuating a cycle of dependence [35–37]. Given the stigma surrounding mental illnesses such as depression in Morocco and other Arab countries, addressing this comorbidity is crucial, especially considering the limited allocation of budgets for mental health services and the shortage of mental health professionals in the region [38, 39].

To effectively support individuals with comorbid depression and SUD in Morocco, it is imperative to utilize valid, reliable, and culturally adapted tools for diagnosing depression. Early identification and intervention can significantly reduce the disease burden and lower the risk of depression [40–43]. A useful tool for assessing depression is the Center for Epidemiologic Studies Depression Scale (CES-D) [44]. The CES-D is one of the most commonly used screening instruments for depressive symptoms [43–45]. The CES-D, which was originally published by Radloff in 1977, is a tool designed to measure the current level of depressive symptoms in general population epidemiological studies and primary care settings. It is a self-report questionnaire consisting of 20 items that have been selected from other validated depression scales. Individual items are reported on a 4-point Likert scale ranging from 0 to 3, where 0 indicates "rarely or none of the time" and 3 indicates "most or all the time" [43, 44]. Scores range from 0 to 60, with higher scores indicating more severe depressive symptoms. The CES-D also offers cutoff scores (such as 16 or higher) that assist in identifying individuals who are at risk of clinical depression with good sensitivity and specificity, as well as high internal consistency [44, 46]. The original factor structure included four factors: depressed affect, positive affect, somatic complaints, and interpersonal difficulties [43, 44].

This measure has been used across age groups, countries, and in both community and institutionalized samples. The initial CES-D testing as well as subsequent sample testing have demonstrated strong psychometric properties as a screening tool [45, 47–49]. It has been one of the most widespread scales for assessing depression since it was published in 1977, and many previous studies support the use of CES-D as a good psychometric test in cross-cultural contexts [43, 50]. According to Shafer, the CES-D is a balanced and comprehensive instrument and is the only instrument that assesses interpersonal aspects widely used as diagnosis criteria for depression (items "feeling that others were unfriendly" and "feeling disliked by others"); however, the other widely used instruments, such as the Beck Depression Inventory (BDI), the Hamilton Rating Scale for Depression (HRSD), and the Zung Self-rating Depression Scale (SDS), do not have such a factor [14, 44, 49]. It is translated into Arabic, Chinese, Dutch, French, German, Greek, Korean, Italian, Japanese, Portuguese, Russian, Spanish, Turkish, and Vietnamese [45]. It is widely used and validated in many clinical and community settings and in different ethnic contexts [43, 49, 51–53], including rheumatoid arthritis, fibromyalgia, and other medical cohorts (stroke, multiple sclerosis, oncology, spinal cord injury, diabetes mellitus); women; diverse populations; primary care; elderly; and clinical and psychiatric populations [45, 54–56].

Although a classical Arabic version of the CESD-R (Center for Epidemiologic Studies Depression-Revised) exists [57], there have been no studies to date that have adapted and validated the CES-D for use in a Moroccan context using standardized methods and in the Moroccan dialect. Transcultural validation studies of depression scales are also rare in Morocco. Given the significance of accurately assessing and preventing depressive symptoms in our Moroccan context, it is crucial to have valid, reliable, and culturally appropriate tools for detecting depression. Because the CES-D is widely used internationally and has demonstrated good psychometric properties, it was deemed valuable to validate a version of the depression rating scale in the Moroccan dialect. The purpose of this study was to conduct a transcultural validation of the English version of the CES-D scale in the Moroccan dialect, using a sample of individuals with substance use disorder in Morocco. The study aimed to assess the reliability and validity of the Moroccan form of the CES-D as a tool for identifying depression.

## Methods

### CES-D translation

The original version of the CES-D was translated from English into a dialectal Arabic. The latter was reviewed by an expert group of psychiatrists, epidemiologists and linguists and finally translated back into English by two independent translators who were unfamiliar with the CES-D. English language specialists reviewed the back translation and made corrections based on their feedback. The final dialectal Arabic version was chosen by the committee after it was judged satisfactory. Thereafter, 20 participants were invited to complete and provide feedback on the scale during a pilot test of the latter. Nothing was found to be unclear or difficult to grasp. Because of this, following the pilot test, no changes were made.

It should be noted that among the problems encountered during cross-cultural adaptation are problems concerning words that did not have a precise equivalent in the Moroccan dialect, problems related to the limited nuances in the expression of the state of mood in the Moroccan dialect, or problems related to the variation in the expression of the state of mood from one region to another. For items that had no equivalent in dialectal Arabic, we had to use sentences that could describe the situation in parentheses next to the term in classical Arabic or use synonyms.

### Participants and procedure

A consecutive series of people who were seeking substance abuse treatment and attending routine follow-up appointments at the addictology center in Fez City were recruited. All participants were informed of the purpose of the study.

Inclusion criteria: being a current user of at least one psychoactive substance, diagnosed with substance use disorder (SUD) by a psychiatrist using the MINI interview, having not yet entered the withdrawal period, being able to communicate and complete the CES-D, in addition to providing demographic information, and agreeing to participate in the study by providing written consent as required by the current legislation.

Exclusion criteria: not being a current user of any psychoactive substance, being in withdrawal, not being able to communicate and complete the CES-D, in addition to providing demographic information, and not agreeing to participate in the study.

### Measures

The CES-D is a 20-item self-report measure used to assess the presence and severity of depressive symptoms in the past week. The items are measured on a four-point Likert scale. Response options range from 0 to 3 for each item (0 = Rarely or None of the Time, 1 = Some or Little of the Time, 2 = Moderately or Much of the time, 3 = Most or Almost All the Time), with a total score between 0 and 60. The original factor structure included four factors: depressed affect (7 items; e.g., feeling lonely or sad, crying spells); positive affect (4 items; e.g., feeling hopeful or happy); somatic complaints (7 items; e.g., decreased appetite, restless sleep, or difficulty getting going); and interpersonal difficulties (2 items; e.g., feeling that others were unfriendly or feeling disliked by others) [43, 44]. Research has consistently found coefficient alphas ranging from 0.68 to 0.92 [58–60].

In phase 1, the 20-item CES-D (original version) was piloted with 140 participants between February 2021 and July 2021. In phase 2, the modified 16-item CES-D (version 2) was distributed to 205 participants between September 2021 and July 2022.

### Statistical analyses

Statistical data analyses were performed on the Jasp and R programs: the exploratory factor analysis (EFA) was performed on the Jasp program 0.17.1 version, and the confirmatory factor analysis (CFA) was performed with the packages "psych", "semTools", and "lavaan" of the R program. The CES-D items in the whole sample were first analyzed by descriptive statistics. Then, the structure and internal consistency of the CES-D were tested. The assessment of factorability was based on the Kaiser–Meyer–Olkin (KMO) test and Bartlett’s sphericity test [61]. The factorial structure of the CES-D was examined on the first sample (*N* = 140) using EFA by principal axis factoring as a method of extraction and oblimin rotation. The selection of the extracted factors was decided on the basis of two different criteria: only factors with an eigenvalue greater than 1 and elements that had a factor loading greater than 0.40 were kept [61–63]. The other items were eliminated. The reliability of the CES-D was assessed based on its internal consistency by determining Cronbach’s alpha coefficient for constructs. The theoretical model of the CES-D was tested by the CFA. A 16-item confirmatory factor analysis was conducted on a sample of 205 participants. The internal consistency was estimated by computing composite reliability (CR), the convergent validity was assessed using the average variance extracted (AVE), and the discriminant validity was tested by the Fornell-Larcker criterion and Hetereotrait-Monotrait ratio [64]. The fitness of the measurement model was determined by RMSEA (Root mean square error of approximation), SRMR (Standardized root mean squared residual), χ2/df (Chi squared value/degrees of freedom), CFI (Comparative fit index), TLI (Tucker-lewis index), and RNI (Relative noncentrality index).

## Results

### Sample characteristics

The study population consisted of two samples of people who were seeking substance abuse treatment at the addictology center in Fez City. The first sample (*N* = 140) was analyzed by exploratory factor analysis, while the second one (*N* = 205) was tested by confirmatory factor analysis. The two samples presented similar demographic characteristics (Table 1). The mean age was 27.81 ± 8.57 (range 15–61) and 29.19 ± 9.85 (range 17–67) for the first and second samples, respectively. 19.30% of the patients in the first sample were married, compared to 17.10% in the second. In terms of the level of education, around 80% of the patients in the two samples had completed secondary school or higher, making up most of the patient population. Most of the patients are male and live in urban and suburban environments.

**Table 1:** Socio-demographic characteristics of the participants

	**Phase 1 (*N* = 140)**		**Phase 2 (*N* = 205)**	
	**Mean (± SD)**	**N (%)**	**Mean (± SD)**	**N (%)**
**Age**				
Female	29.57 (± 7.54)		29.00 (± 8.89)	
	(Range 17- 43)		(Range 17- 44)	
Male	27.61 (± 8.68)		29.21 (± 9.99)	
	(Range 15—61)		(Range 17- 67)	
Total	27.81 (± 8.57)		29.19 (± 9.85)	
	(Range 15—61)		(Range 17- 67)	
**Gender**				
Female		14 (10.00)		25 (12.20)
Male		126 (90.00)		180 (87.80)
**Marital status**				
Married		27 (19.30)		35 (17.10)
Unmarried		105 (75.00)		155 (75.60)
Divorced		8 (5.70)		15 (7.30)
Widowed		0 (0.00)		0 (0.00)
**Education**				
Illiterate		3 (2.10)		5 (2.40)
Primary education		25 (17.90)		34 (16.60)
Secondary education		85 (60.70)		119 (58.00)
Higher Education		27 (19.30)		47 (22.90)
**Living environment**				
Rural		8 (5.70)		12 (5.90)
Urban		118 (84.30)		170 (82.90)
suburban		14 (10.00)		23 (11.20)

### Exploratory factor analysis

The underlying factor structure of the CES-D was examined by analyzing the data from the first sample (*N* = 140). The sampling adequacy for performing the analysis was verified through the Kaiser–Meyer–Olkin test. The total KMO value was 0.90, suggesting excellent sampling adequacy. Bartlett’s test of sphericity (χ2 = 2339.045; df = 190, *p* < 0.001) indicated that inter-item correlations were sufficiently large to perform EFA.

The selection of the extracted factors was decided based on two criteria: only factors with an eigenvalue greater than 1 and elements that had a factor loading greater than 0.40. Items that failed to load higher than this threshold were eliminated, and for this reason, items 2, 7, 10, and 20 have been eliminated, and three factors were extracted, with an explained variance of 78.9% (Table 2).

**Table 2:** Factor structure of the dialectal Arabic version of the CES-D (16 items)

**Short item names**	**Components of the factor**			**Uniqueness**
	**Somatization**	**Interpersonal difficulties/Emotional vulnerability**	**Positive affect**	
Depressed	0.92			0.14
Sad	0.90			0.14
Bothered	0.89			0.23
Blues	0.88			0.21
Sleep	0.84			0.30
Failure	0.82			0.32
My mind	0.80			0.40
Disliked		0.94		0.14
Unfriendly		0.93		0.13
Cry		0.89		0.19
Talked less		0.87		0.25
Lonely		0.87		0.24
Hopeful			0.93	0.16
Enjoy			0.92	0.16
As good			0.91	0.17
Happy			0.87	0.21
Eigenvalue	5.25	4.06	3.31	
Variance (Total = 78.9%)	32.8%	25.4%	20.7%	

The first factor labeled "Somatization", with an explained variance of 32.8%, was loaded with 7 items referring to mixed affective and somatic symptoms. The name "somatization" given to this factor" is the same name used by Iwata and Roberts (1996) for the combination of somatic and depressive symptom items [65]. The second factor labeled "Interpersonal difficulties/Emotional vulnerability", with an explained variance of 25.4%, was loaded with 5 items related mainly to interpersonal difficulties (e.g., disliked, unfriendly, lonely, talked less); and the third factor "positive affect", with an explained variance of 20.7%, was loaded with 4 items referring to well-being, the same items contained in the positive affect factor of the original version.

#### Internal consistency

The most commonly used measures of internal consistency are Cronbach's alpha and CR, which measure reliability based on observed item correlations. The alpha values need to be at least 0.70 and ideally above 0.80 to be considered good consistency [61]. The reliability of the CES-D was assessed based on its internal consistency by determining Cronbach’s alpha coefficient and CR (Table 3). The subscales had alpha values between 0.88 and 0.93 and CR values between 0.89 and 0.93, which confirmed very good internal consistency. This means that all constructs were reliable.

**Table 3:** Composite reliability, average variance extracted, and correlations between constructs

**Latent constructs**	**Alpha** *(Total* = *0.90)*	**CR**	**AVE**	**Latent constructs**		
				1	2	3
**1.** Somatization	0.93	0.93	0.64	**0.80**		
**2.** Interpersonal difficulties/ Emotional vulnerability	0.91	0.91	0.66	0.57^a^	**0.81**	
**3.** Positive affect	0.88	0.89	0.66	0.26^b^	0.19^c^	**0.81**

*CR* Composite reliability, *AVE* Average variance extracted

^a^*p* = *0.0001*

^b^*p* = *0.002*

^c^*p* = *0.019*

### Confirmatory factor analysis

#### Convergent validity

**Fig. 1:**
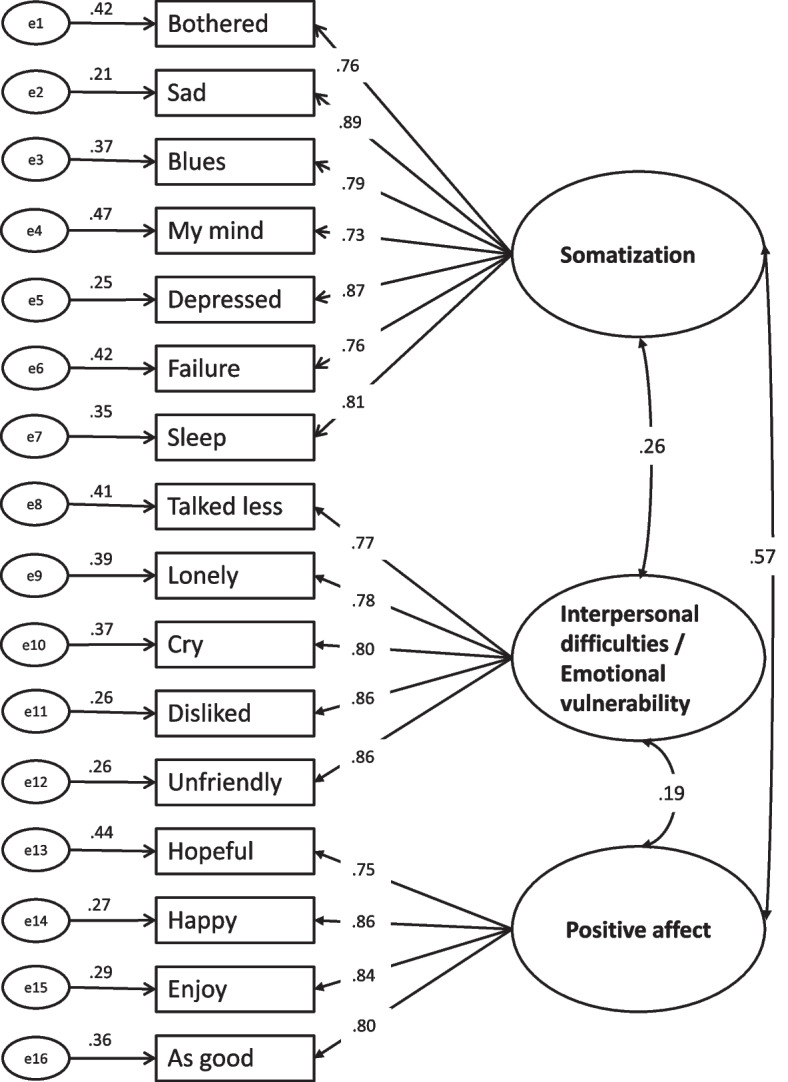
CFA measurement model

Convergent validity is an assessment for measuring the level of correlation between multiple agreed-upon indicators of the same structure. To determine convergence validity, the CR and the AVE should be considered [66, 67]. The values range from 0 to 1. The AVE value should be greater than 0.50, and the CR should be comprised of 0.7 and 0.95, which is sufficient for convergence validity [66–69]. The high values of the CR (0.89–0.93) and AVE (0.64–0.66), respectively, showed satisfactory convergent validity of the CFA measurement model (Fig. 1).

#### Discriminant validity

Discriminant validity refers to the extent to which the constructs differ from each other empirically. It also measures the degree of difference between overlapping constructs [67]. Discriminant validity can be assessed by the Fornell & Lacker criterion, and the HTMT [67, 68].

The bolded values are the square root of AVE of each dimension, whereas the other values are the inter-correlation among the latent factor dimensions (Table 3). The highest correlation value between factors was 0.57 (between Somatization and Interpersonal difficulties/Emotional vulnerability), while the smallest value among the square root of AVE values was 0.80. The matrix diagonal values were higher than the off-diagonal values in the corresponding rows and columns [64]. The HTMT value should be less than 0.85 or 0.90 [66, 70, 71].

It appears from Table 4 that all matrix values are below 0.85. The findings warranted discriminant validity between all constructs of the proposed model. Overall, both reliability and the two types of construct validity tests (convergent and discriminant validity tests) showed that the proposed measurement model construction was justified for at least these two types of tests (Fornell and Larker criterion and HTMT).

**Table 4:** Discriminant validity analyses: Heterotrait-Monotrait (HTMT) Criterion results

Latent constructs	Latent constructs		
	**1**	**2**	**3**
**1.** Somatization	1.00		
**2.** Interpersonal difficulties/Emotional vulnerability	0.55	1.00	
**3.** Positive affect	0.22	0.16	1.00

#### Fitness of the measurement model

The fit statistics for the CFA model were χ2 = 187.39, CFI = 0.96, TLI = 0.95, RNI = 0.96, RMSEA = 0.06, SRMR = 0.04, and χ2/df = 1.86 (Table 5). These goodness-of-fit measures were acceptable when following the threshold values for fit statistics: the χ2/df should be less than 3, CFI and TLI should be greater than 0.95, RNI should be greater than 0.90, the RMSEA should be less than 0.07, and the SRMR should be less than 0.08 [61, 72, 73]. Based on these ranges, all values were within acceptable thresholds. Therefore, the measurement model showed an adequate to a good fit.

**Table 5:** Overall fit indices of the CFA model

Fit index	χ2/df	SRMR	RMSEA	CFI	TLI	RNI
Observed Value	1.86	0.04	0.06	0.96	0.95	0.96
Level of acceptance	< 3	< .08	< .07	> .90	> .90	> .90

*χ2* Chi-squared value, *df* degrees of freedom, *SRMR* Standardized root mean squared residual, *RMSEA* Root mean square error of approximation, *CFI* Comparative fit index, *TLI* Tucker-lewis index, *RNI* Relative noncentrality index

## Discussion

The main objective of this study was to create a Moroccan dialectal Arabic version of the CES-D that is applicable in the local context. Two groups of 140 and 205 individuals who were seeking treatment for substance abuse at the Addictology Center in Fez City were recruited for the study. Most of the patients are males who reside in urban or suburban areas. These sociodemographic characteristics can be explained by the fact that men are more prone to using various types of illicit drugs [74], and the addiction treatment center is located in an urban setting.

In our study, the factor analysis of the CES-D yielded three-factor solutions with a combined affective and somatic factor. This factor structure is different from the originally reported four-factor structure; many studies have confirmed this four-factor structure of the CES-D [75–86]. Our finding was similar to the three-factor structure described among other ethnic groups, including Chinese, Spanish, Arabic, American-Indian, and American-African origins, where the original four-factor solution was not replicable [87–94]. While others reported a two-factor model with depressive, somatic, and interpersonal items loading on a single factor and positive affect items on another [86, 95–98], or even five factors [99] or one factor [100].

The three-factor structure found indicates that our participants have both affective and somatic expressions for conveying depressive feelings. In the Arab world, our three-factor structure is similar to the structure found by Ghubash et al. (in 1992 and 2000) in two samples of Arab women [87, 101]. In support of this, Dardas et al. (2016) found that Arabs tend not to interpret cognitive symptoms of depression, such as feelings of worthlessness and preoccupation with death, and physical symptoms of depression, such as fatigue and insomnia, as mood symptoms. In line with these findings, it should be noted that variation in the somatization domain is considered one of the most consistent findings in cross-cultural studies of depression. In particular, non-Western countries report a greater emphasis on the physical component [102–106]. These results support the existing evidence that highlights the significant influence of ethnicity and culture on factor structure. The findings also suggest that there are clear differences in the experience of depressive, somatic, and interpersonal symptoms between Western and non-Western countries. There is evidence suggesting that in Western cultures (e.g., American), depression may be driven by psychological factors, while in Eastern cultures (e.g., Chinese and Korean), it may be driven by somatic factors [107]. Western cultures have been found to be more proficient in distinguishing between psychological, somatic, and interpersonal symptoms of depression when compared to Asian and Arabic populations [25, 28–30, 108–111]. People in Western cultures are often believed to place too much emphasis on distinguishing between depressive symptoms of the mind and body, with a particular focus on the emotional or psychological aspects [108].

In support of these findings, Uluşahin et al. conducted a cross-cultural study on depressive symptoms among outpatient samples in both British and Turkish populations. In the Turkish sample, the first common component responsible for the greatest variability was the somatization factor, while in the British sample, it was the component that reflected fundamental depressive symptoms [112]. In his report, Radloff utilized principal component factor analysis with varimax rotation and identified four distinct factors that were interpretable: depressed affect, positive affect, somatic and retarded factor, and interpersonal factor. These factors accounted for 48% of the variation. Using a similar method, Kuo (1984) identified three factors, namely depressed and somatic, interpersonal and positive affect, that account for 53% of the variance [95]. Therefore, relying solely on standardized tools developed by or for Western cultures can result in errors in assessment and inaccurate estimates of psychopathology. Especially considering the absence of a universal understanding of mental disorders, including depression.

In our study, we obtained a Cronbach's alpha of 0.90 for the 16-item scale derived from CFA, with alphas ranging between 0.88 and 0.93 for the three factors extracted from this analysis. These findings demonstrate excellent internal consistency for the scale, indicating high reliability. Furthermore, the CES-D scale has consistently shown good reliability across various sociocultural contexts. For example, Brett D Thomb et al. (2008) found an alpha of 0.88 [113], α = 0.85 [114] for Ghazali et al. (2016), α = 0.88 [89] for Heo et al. (2018), α = 0.84 [115] for Nathaniel Chishinga et al. (2011), α = 0.92 [116] for Barnabas K Natamba et al. (2014), α = 0.88 [41] for Dardas et al. (2019), α = 0.84 for [117] Logsdon and Myers (2010), α = 0.83 [118] for Aebi et al. (2009), α = 0.90 [119] for Yang et al. (2004), α = 0.85 [120] for Roberts (1980), α = 0.85 [121] for Himmelfarb and Murrell (1983), α = 0.89 [122] for Chon and Rhee (1992), α = 0.80 [123] for Shin et al. (1991).

The three-factor measurement model demonstrated an excellent to satisfactory fit. CFA measures were used to assess the validity and reliability of the features. However, the validity of the measurement operation is severely restricted due to the location, timing, and utilization of the scores, particularly in studies with small sample sizes. Results from research conducted in one location with a specific population may be challenging to generalize to another sample from a different location and/or demographic.

## Conclusions

Overall, this study is the first time that a dialectal Arabic CES-D version has been validated among Moroccans with SUD. We investigated its psychometric properties among a sample of 205 Moroccans with SUD using CFA to examine its factor structure. However, this study has some limitations that should be highlighted. The sampling was conducted in a single addictology center and targeted a small sample size of patients with SUD. In addition, the sample is small and contains patients of different socio-demographic characteristics. Hence, these findings cannot, however, be extrapolated to all Moroccan patients with substance disorders from different regions. Therefore, additional research on larger samples of various populations, as well as longitudinal surveys, are needed to evaluate the scale's predictive validity for psychosocial outcomes. This version of 16 items is a quick, valid, and reliable instrument to identify depressed people or those at risk for developing depression, to allow for intervention, prevent, and/or treat depression early in order to decrease disease burden, and decrease the risk of depression.

## Data Availability

The datasets used and analyzed during the current study are available from the corresponding author on reasonable request.

## Abbreviations

AVE: Average variance extracted
BDI: Beck Depression Inventory
CES-D: Center for Epidemiologic Studies Depression Scale
CFA: Confirmatory factor analysis
CFI: Comparative fit index
CR: Composite reliability
DALYs: Disability-adjusted life years
DSM-V: Diagnostic and Statistical Manual of Mental Disorders, 5th Edition
EFA: Exploratory factor analysis
HCP: Haut Commissariat au Plan
HRSD: Hamilton Rating Scale for Depression
HTMT: Heterotrait-Monotrait criterion
KMO: Kaiser-Meyer-Olkin
RMSEA: Root mean square error of approximation
RNI: Relative noncentrality index
SDS: Zung Self-rating Depression Scale
SRMR: Standardized root mean squared residual
SUD: Substance use disorders
TLI: Tucker-lewis index
WHO: World Health Organization
χ2/ df: Chi squared value/degrees of freedom
